# A Case Report on Methimazole-Induced Severe Hypothyroidism

**DOI:** 10.7759/cureus.21339

**Published:** 2022-01-17

**Authors:** Mehrdad Alaie, Amanda Tramutola, Daniel Mukamal

**Affiliations:** 1 Emergency Medicine, St. Barnabas Hospital Health System, Bronx, USA

**Keywords:** covid-19, amiodarone, methimazole, polypharmacy, hypothyroid, myxedema crisis

## Abstract

Severe, uncorrected hypothyroidism can result in a dangerous hypometabolic state leading to myxedema crisis, a rare but life-threatening illness. Myxedema crisis is a clinical diagnosis. The treatment for severe hypothyroidism and myxedema crisis is multifaceted, centered around thyroid hormone replacement, identification and treatment of precipitating factors, and intricate supportive care. Even with early and aggressive treatment, the myxedema crisis carries a high mortality rate. We present a case of iatrogenic hypothyroidism with severe features in a patient concurrently taking methimazole and amiodarone. This case illustrates the need for a high index of suspicion for patients on treatment for hyperthyroidism with any inciting event. The complexity of this case was rooted in polypharmacy, specialized care for numerous comorbidities complicated by significant strain on ED resources and personnel due to a global pandemic, resulting in severe complications. This case brings to light the need for immediate and appropriate treatment and critical care consultation for admission to a unit with an advanced level of care, as is the case for all patients with concern for severe hypothyroidism or myxedema crisis.

## Introduction

Hypothyroidism itself is a relatively common endocrine disorder, affecting approximately one to five percent of adults in the United States and up to 15 percent of those over 75 years of age [1]. Patients may present with symptoms of fatigue, muscle cramps, cold intolerance, and shortness of breath and may have physical exam findings consisting of periorbital edema, bradycardia, macroglossia, hypoventilation, nonpitting peripheral edema, and cool, dry skin. Hypothyroidism can progress to myxedema crisis in the setting of any major insult to the body, including infection, trauma, ischemic events, or exacerbation of congestive heart failure or chronic obstructive pulmonary disease [1]. Myxedema crisis is an extreme hypometabolic state resulting from severe, uncorrected hypothyroidism. While insufficient thyroid hormone leads to slower cell metabolism in general, the multiorgan dysfunction characterized by myxedema crisis occurs when the bioavailability of thyroid hormone reaches a critical low [1]. Various medications can precipitate this condition as well, spanning such classes, including sedatives, narcotics, beta-blockers, and antithyroid agents.

A patient in a true myxedema crisis should display characteristic physical exam findings consisting of bradycardia, hypotension, hypothermia, hypoventilation, and altered mental status. These patients will likely develop hypoglycemia as well, which is believed to occur due to decreased gluconeogenesis, decreased insulin clearance, and coexistent adrenal insufficiency [1]. The treatment for severe hypothyroidism or myxedema crisis is multifaceted, centered around thyroid hormone replacement, identification and treatment of precipitating factors, and immediate supportive care. Even with early and aggressive treatment, myxedema crisis carries a mortality rate of 30 percent or more depending upon a patient’s comorbid conditions and age. The persistence of bradycardia and hypotension serve as additional indicators of a poor prognosis [2]. All patients presenting to the emergency department with a suspected diagnosis of myxedema crisis warrant immediate treatment without confirmatory testing, followed by intensive care unit admission.

## Case presentation

A 68-year-old female presented to the emergency department via ambulance, her chief complaint being “feeling shaky and weak for about a month”, with tingling and cramping sensations in her hands and feet. The patient had a documented medical history of hypertension, congestive heart, coronary artery disease with the placement of a drug-eluting, atrial fibrillation with an automated implantable cardioverter-defibrillator in place following a failed ablation, chronic obstructive pulmonary disease requiring home oxygen via nasal cannula, bipolar disorder, dementia, hyperthyroidism, and recent COVID-19 infection. Relevant home medications include amiodarone 200 mg daily, methimazole 2.5 mg daily, sodium bicarbonate 650 mg twice a day, rivaroxaban 20mg daily, metoprolol 50 mg daily, and spironolactone 25 mg daily.

The patient was found to be hypoglycemic to 29 milligrams per deciliter (mg/dL) (reference range: 70-99 mg/dL) and was subsequently provided 25 grams of dextrose intravenously. Upon her arrival to the emergency department, the patient was found to be hypotensive to 78/51 millimeters of mercury (mmHg) with a heart rate of 66 beats per minute (bpm).

**Figure 1:**
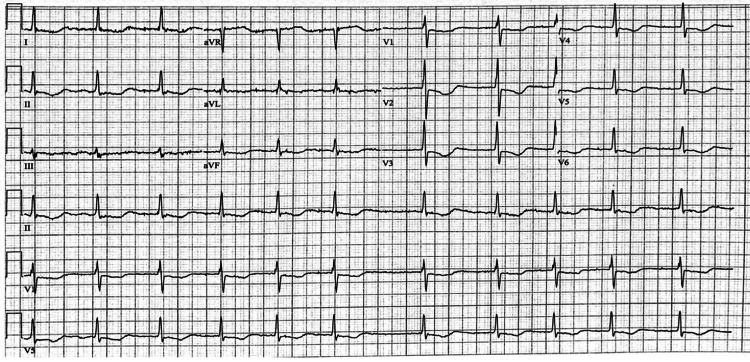
Patient's electrocardiogram, demonstrating rate-controlled atrial fibrillation with t-wave inversions in the precordial leads

Performed electrocardiogram demonstrated rate-controlled atrial fibrillation with t-wave inversions in the precordial leads (see Figure 1).

Her oral temperature was 37 degrees Celsius, and her oxygen saturation was appropriate on her home dose of supplemental oxygen. She was again found to be hypoglycemic to 54 mg/dL shortly after her first dextrose bolus and was provided another 25 grams of dextrose intravenously. She was awake and alert but was not fully oriented, though given her history of dementia, it was unclear if this deviated from her baseline mental status. Her lungs were clear to auscultation, her heart rhythm was irregular at a normal rate without murmurs, and her abdomen was soft and non-tender. She was noted to have mild periorbital edema, slight bilateral ptosis, and moist mucous membranes. Her speech was clear and fluent. Peripherally, her extremities were dry and cool but acyanotic, and nonpitting pretibial edema was appreciated to her lower legs. She did not display any gross signs of trauma or gross neurologic deficits, and she did not appear to be in acute distress. Initial interventions included intravenous administration of both glucose and normal saline, both of which had only transient effects. At this point, the patient was received as a sign-out to the next team during a very busy shift.

An initial series of laboratory tests were performed, which revealed a multitude of significant findings, including a potassium level of 2.1 milliequivalents per liter (mEq/L; reference range: 3.5-5.3 mEq/L), which was aggressively repleted, a sodium level of 130 mEq/L (reference range: 135-145 mEq/L), a thyroid-stimulating hormone level of 15.46 international units per milliliter (u[IU]/mL; reference range: 0.34-5.60 u[IU]/mL), and a total T4 level of 3.3 micrograms per liter (ug/L; reference range: 6.0-12.0 ug/L). The patient was also found to have a mild leukocytosis with leftward shift, a moderate acute kidney injury, and mild transaminitis. These results, in conjunction with the patient's physical exam findings and vital signs, suggested the presence of a significant hypothyroid state. The patient was provided levothyroxine 100 micrograms and hydrocortisone 100 milligrams intravenously and shortly thereafter reported a considerable improvement in her symptoms and blood pressure (BP 115/56). This patient's blood glucose levels (105 mg/dl) began to normalize as well, and she became more alert, answering questions appropriately, and remained stable throughout her stay in the emergency department. The patient was subsequently admitted to the intensive care unit. Of note, while initial free T3 level (1.9 picograms per milliliter [pg/mL], reference range: 2.0-4.4 pg/mL) and free T4 level (1.1 nanograms per deciliter [ng/dL], reference range: 0.82-1.77 ng/dL) were within normal limits, these results are likely skewed because they were drawn after the administration of levothyroxine. For reference, the patient had routine thyroid function testing performed about five weeks earlier, with results including a thyroid-stimulating hormone (TSH) of 18.52 IU/mL, a total T4 of 5.5 ug/L, a free T3 of 2.6 pg/mL, and a free T4 of 0.88 ng/dL.

Her inpatient stay was largely unremarkable aside from a detailed evaluation by the endocrinology service. Subsequent testing did not indicate an active autoimmune process, and a morning cortisol level of 14.2 ug/dL helped to rule out adrenal insufficiency (reference range 5-25 ug/dL). She remained stable and asymptomatic during her hospital course while being treated with thyroid hormone supplementation and was discharged home in stable condition. Methimazole was discontinued, and the patient was discharged on low-dose levothyroxine, with plans for endocrinology follow-up.

## Discussion

Methimazole is a relatively common medication used in the long-term management of hyperthyroidism. Part of a class of medications known as thionamides, methimazole functions by both altering the availability of iodine as necessary for thyroid hormone synthesis as well as preventing the final hormone product from forming into a functional substance [3]. As a treatment for hyperthyroidism, methimazole can be implicated as a cause of iatrogenic hypothyroidism under certain circumstances.

Amiodarone, also taken by our patient, is another known iatrogenic cause of thyroid disease, exhibiting effects largely dependent upon a geographical location. In iodine-deficient regions, amiodarone is more likely to precipitate thyrotoxicosis. In contrast, amiodarone-induced hypothyroidism (AIH), which is more common overall, is more often seen in iodine-sufficient populations, with Hashimoto's thyroiditis being the most common risk factor [4]. The mechanism of action for AIH is two-fold. First, amiodarone includes a high measure of iodine, which can lead to loss of normal thyroid autoregulation [5]. Second, amiodarone has several intrinsic effects on thyroid hormone functionality, including inhibiting the conversion of T4 to the more active T3, inhibition of T4 and T3 entry into peripheral tissue, and increasing accumulation reverse T3 (rT3) by decreasing clearance. rT3 is the metabolically inactive form of thyroid hormone, and in turn, facilitates a hypothyroid state. In addition, amiodarone is cytotoxic to thyroid follicular cells and can lead to thyroiditis [5,6,7].

Treatment of AIH primarily consists of levothyroxine administration (especially for overt forms, defined as elevated TSH that is >10 mU/L with a low free T4). The goal for levothyroxine therapy is to normalize serum levels of free T4 and free T3, and thus thyroid hormone replacement may not be necessary for patients with subclinical forms of AIH (defined as TSH <10 mU/L but greater than the upper limit of the normal reference range, with a normal free T4). The decision to treat should include careful consideration of the risks of adverse cardiac events due to overtreatment. Fifty percent of AIH cases in patients without underlying thyroid pathology will resolve naturally within two to three months [4]. However, in cases of patients with underlying Hashimoto's thyroiditis, they are more likely to sustain persistent hypothyroidism [6] and may need continued treatment. In both overt and subclinical forms of AIH, amiodarone can, in fact, be safely continued [4].

In our patient's case, the concomitant use of methimazole and amiodarone likely precipitated her hypothyroid state. This case adds to the current literature of a rare drug interaction of methimazole, a medication that is thought to be fairly safe in the treatment of hyperthyroidism. Our patient, whose presentation was consistent with severe hypothyroidism, highlights the importance of physical exam findings to help reach a prompt diagnosis and initiate emergent treatment. If there is any concern for myxedema crisis, treatment should not be delayed by waiting for confirmatory laboratory results [2]. Our patient's characteristic physical exam findings suggested a hypothyroid state which was subsequently confirmed with her thyroid function tests. However, had she presented in extremis, she would have benefitted from emergent treatment without regard for test results.

Another possible contributor to our patient's presentation could be due to her recent recovery from the COVID-19 infection. While studies are still ongoing, they show a clear contribution of COVID-19 on thyroid dysfunction. Autopsies of deceased COVID-19 patients show thyroid follicular cell damage with no viral particles found in the thyroid tissues, indicating that pathogenesis seems to involve indirect immune-mediated destruction of the thyroid [8]. Although hyperthyroidism is more commonly manifested, hypothyroidism is also seen in a significant cohort of these patients, with a positive correlation between the degree of thyroid dysfunction and severity of COVID-19 disease [9]. Thyroid function testing thus becomes especially important as severe dysfunction in these lab values may become prognostic of critical-care level COVID-19 patients. As the COVID-19 pandemic continues to remain at the forefront of the current medical climate, this indicator may be an important tool for clinicians to keep in mind.

It is also worth noting that this case had been signed out to the next shift prior to a diagnosis having been established, emphasizing the significance of viewing sign-outs as a fresh perspective to each case. Sign-out communication and the transition of care are notoriously viewed as a dangerous time for patient care. In our patient's case, her labs had been carefully evaluated, and her hypothyroid state had been identified and properly treated in a timely manner by the oncoming team. Had this not been the case, she may have required unnecessary treatments (for example, central line placement and vasopressor initiation) or had worsened outcomes. This is not to disparage the work of the primary team, who initially evaluated and stabilized the patient. However, it is important to re-evaluate each case by obtaining a new detailed history and physical examination, carefully reviewing the previous workup, repeating vital signs, and avoiding biases such as anchoring. This case underlines the importance of taking every opportunity to re-assess a critically ill patient while embracing the collaborative nature of emergency medicine.

## Conclusions

In summary, this is the case of severe iatrogenic hypothyroidism induced by polypharmacy in the midst of a global pandemic. A rapid escalation of therapeutic treatment resulted in substantial and favorable outcomes, as did quick recognition of the underlying pathology by the incoming physicians after change-of-shift. A thorough review of the patient's prior records and medication review helped identify a likely drug interaction as the cause of her symptoms. This led to proper management and favorable outcomes with a short hospital course and appropriate outpatient follow-up.
